# Developing and refining behaviour-change messages based on the Brazilian dietary guidelines: use of a sequential, mixed-methods approach

**DOI:** 10.1186/s12937-020-00585-1

**Published:** 2020-07-06

**Authors:** Neha Khandpur, Priscila de Morais Sato, Jose Ribeiro Gouveia Neto, Fernanda Scagliusi, Patricia Constante Jaime

**Affiliations:** 1 Department of Nutrition, School of Public Health, University of São Paulo, Av. Dr. Arnaldo, 715-Cerqueira César, São Paulo, 01246-904 Brazil; 2 Center for Epidemiological Studies in Health and Nutrition (NUPENS), Faculty of Public Health, University of São Paulo, Av. Dr. Arnaldo, 715-Cerqueira César, São Paulo, 01246-904 Brazil; 3 Department of Nutrition, Harvard T.H. Chan School of Public Health, 677 Huntington Avenue, Boston, MA 02115 USA

**Keywords:** Behaviour-change messages, Dietary guidelines, Mixed-methods, Brazil

## Abstract

**Background:**

Dietary Guidelines are an important tool for population health promotion efforts. However, current surveillance data suggest that only a small minority of the population meet the 2014 Brazilian Dietary Guidelines (BDG) recommendations. Translating recommendations into practice may not be immediately clear and behavior-change messages guiding the behaviors that need to be changed and identifying substitute practices to meet a specific recommendation, are required. This study details the methods undertaken to develop and refine messages supporting the adoption of healthy dietary choices and behaviors in adults, as outlined in the BDG.

**Methods:**

A sequential, five-step, mixed-methods approach, determined a priori, was followed for designing and refining messages. These included: (1) content extraction; (2) audience analysis; (3) input from an expert review panel; (4) message development and message refinement; and a (5) test of content validity.

**Results:**

The content extraction process led to the identification of 63 excerpts from the BDG, organized into themes. The audience analysis highlighted barriers to healthy eating that included lack of time (to eat, to cook), difficulty in accessing healthy food, the convenience and the ubiquitous marketing of ultra-processed foods. Twenty of the 63 DG excerpts reviewed by the expert panel were identified as being a priority for message development and total of 111 messages were developed. Messages were short, structured to be one-sided, conveyed the most important information at the beginning (anticlimactic), used simple language and were explicit in the information they relayed. They were positive and gain-framed and used an empathetic, solution- or substitution-based tone and were presented in the active voice. The messages focused on goals and skill development, behavior regulation, incentivized positive practices as time and/or cost saving. Content validity testing helped further messages and reduced the number of messages from 111 to 40.

**Conclusions:**

This study provides the blue-print for the phase-wise development of messages that synthesize the key recommendations of the food-based BDG and communicate the adoption of behaviors and goals that are consistent with it’s message. It details methods which could be adapted and replicated for message development in other contexts.

## Contributions to the literature

This manuscript:

1. Addresses the dearth of evidence on translating dietary guidelines into actionable and accessible behavior-change messages, furthering implementation science.
2. Details the five-step, mixed-methods approach undertaken to craft and refine theory-based messages supporting the adoption of healthy dietary practices, as outlined in the 2014 Brazilian Dietary Guidelines.
3. Includes results and examples of progressive message refinement and discusses how the methods could be contextually adapted.
4. The final set of messages help distinguish between what to eat from how to incorporate that guidance and are more likely to make dietary change efforts successful.

## Introduction

Food-based Dietary Guidelines are an important tool for population nutrition education and health promotion efforts [1]. They detail the most recent scientific evidence on the relationship between diet and health and provide advice on healthy food choices for preventing country-specific, diet-related non-communicable diseases (NCDs). The Dietary Guidelines serve as the foundation for nutrition education material, the basis of federal feeding programs, school meals, procurement standards, and inform programs, policies and communication efforts [2–4]. Like other Dietary Guidelines (DG), the 2014 Brazilian Dietary Guidelines (BDG) present evidence-based recommendations for the population on food choices and the avoidance of industrialized, ultra-processed foods but go further in their coverage of topics ranging from the introduction of the NOVA classification (a system that categorizes food based on their purpose and extent of processing) meal structures, and modes of eating to presenting strategies for overcoming barriers to healthy eating [5]. The BDG account for the social, cultural, economic and environmental implications of dietary patterns and are part of Brazil’s concerted effort to address the modifiable lifestyle factors associated with NCDs [6].

Current dietary patterns and nutrition behaviors suggest that only a small minority of the Brazilian population meet BDG recommendations. Instead of being completely avoided, ultra-processed food intake is high at 30% of total caloric consumption in Brazil and is associated with overweight and obesity in these populations [7]. Eating out is not uncommon - Brazilians spent 25% more of their food budget on meals away from home between 2002/03 and 2008/09 [8]. This a cause for concern given the lower nutritional quality of meals prepared outside the home [9]. Only 29.9% of the population reports consuming fruit and vegetables on five or more days of the week and even fewer (18.2%) meet the recommended intake of 5 portions per day [10]. Almost twice as many eat high fat foods and 77% consume sugary drinks at least once a week, with more regular consumption (five or more days of the week), reported in 28% [10]. Effective strategies to more widely disseminate the DG will be needed if Brazil is to meet its Strategic Action Plan Goal by 2022, of increasing fruit and vegetable consumption, reducing the average salt consumption and decreasing the prevalence of obesity in children, adolescents and adults [11].

The expert knowledge-consumer behavior gap suggests that DG need be made more accessible, and actionable to be potentially more effective. In addition to elaboration and publication, effective actions for translating the DG recommendations are crucial. While freely available online, and disseminated through social media sites of the Ministry of Health and the Unified Health System, it is unlikely that the DG are widely or frequently accessed by the general population who may find the 5-chapter, 158-page, technical report dense and long, or who may need additional support before they attempt to act on its recommendations. For most people, it may not be immediately clear what series of behaviors need to be changed and what the substitute practices are, to meet a specific recommendation. Indeed, research suggests that distinguishing between dietary guidance (what to eat) from nutrition promotion (how to incorporate that guidance) is more likely to be successful in promoting dietary change [12]. Therefore, distilling the BDG into actionable messages with an explicit behavioral component would be the first, necessary step in making the guidelines more feasible to implement for the general population.

While the importance of creating practical messages has been previously emphasized [13] and best practices for creating messages outlined [14], few studies have documented the process of designing behavior change messages based on DG [15–17] or made explicit the methods used [18, 19]. Messages have been a crucial part of health communication campaigns or behavior-change interventions but the process of message development, refinement and testing has rarely been reported in the literature [20]. This lack of evidence means there is no way of determining whether message-design was based on rigorous, reproducible methods or plain intuition. Past work has also focused on one or two specific behaviors and few have looked at changing a set of practices as targeted by the BDG. This present study seeks to further the evidence in implementation science by presenting the methods undertaken to develop and refine messages supporting the adoption of a variety of healthy dietary choices and behaviors in adults, as outlined in the 2014 Brazilian Dietary Guidelines.

## Methods

**Fig. 1:**
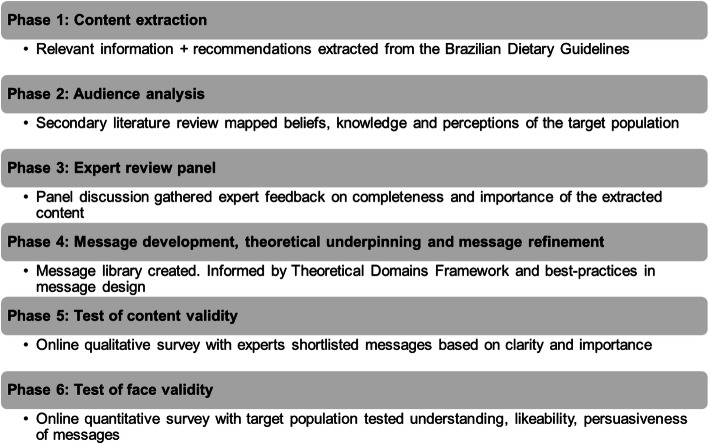
The sequential, mixed-methods approach used for designing and refining messages that support healthy behaviors, based on the 2014 Brazilian Dietary Guidelines

A sequential, five-step, mixed-methods approach, determined *a priori*, was followed for designing and refining messages that support behaviors and choices consistent with the BDG recommendations (see Fig. 1). These messages were designed to be applicable to a wide demographic and were aimed at urban adults. This research methods included:

1. Step 1: Content extraction
2. Step 2: Audience analysis
3. Step 3: Expert review panel
4. Step 4: Message development, theoretical underpinning and message refinement
5. Step 5: Test of content validity

Step 6 tested the face validity of the messages with the target audience. Results from this phase of the study are reported in a subsequent article.

The study was granted ethical approval on the 27th of September 2017 by the Ethics Committee of the University of São Paulo (69,039,117.0.0000.5421) and data was collected between October 2017 and August 2018. This study was conducted on behalf of the Brazilian Ministry of Health. The methodological approach was informed by previous literature but also took into consideration time and financial constraints.

### Step 1: Content extraction

Content extraction was fundamental for identifying excerpts from the BDG that would make appropriate material for the messages. The BDG content broadly encompasses guidelines/recommendations for a healthy diet along with quantitative and qualitative evidence to justify why these guidelines are important to follow. For this exercise, only recommendations from the BDG were extracted. Two researchers (NK and PdMS), working independently, read through all chapters of the BDG to identify, extract and record relevant sections, verbatim, into an Excel spreadsheet along with information on the chapter and the themes that the excerpt addressed. The two extraction sheets were compared to include any missing information, remove redundancies and to compile a final list of relevant content.

### Step 2: Audience analysis

A formative literature review was undertaken to map beliefs, knowledge and perceptions of adult Brazilians living in urban and semi-urban settings, with regards to healthy eating. Awareness, current dietary patterns, perceived barriers and opportunities towards healthy eating were also assessed. The aim of this step was to better understand the target population of these messages and use this information to design the messages that informed them about healthy practices and provided justification for adopting these practices while countering misconceptions. The databases LILACS, FSTA, Ebsco, SCIELO and Pubmed were searched from their inception to October 2017 for quantitative and qualitative articles published in either English or Portuguese using the key words *food practices*, *beliefs about food*, *beliefs about processed food*, *attitudes towards food*. The search was conducted by a study researcher (JRGN) and was geographically restricted to Brazil. A narrative review was conducted to summarize the findings.

### Step 3: Expert review panel

A panel of experts was invited for a three-hour discussion to help: (i) determine the completeness of the extracted BDG content, (ii) assess the hierarchy of importance of the content, (iii) provide feedback on results from the audience analysis, and (iv) share suggestions for organizing and presenting messages. The four-member panel was composed of researchers who were part of the scientific committee tasked with the development of the original BDG. The experts studied the socio-cultural dimensions of eating practices, and had previously developed various interventions and data collection instruments based on the BDG and therefore were extremely familiar with its content. Study objectives and results from the audience analysis were presented by the study team at the start of the session along with a list of the content extracted from the DG. Experts first recorded their opinions on the relative importance of the content and followed that up with a more detailed discussion to elaborate on their opinions, highlight new information to be included and illustrate ways to contextualize the messages that incorporated learnings from the audience analysis. The discussions were audio-recorded and JRGN served as the notetaker, capturing all suggestions made and decisions reached.

### Step 4: Message development, theoretical underpinning and message refinement

Steps 1–3 set the foundation for the creation of a message library that broadly aimed at improving access and comprehension of the BDG and encouraging and facilitating behavior change. Messages were designed to improve awareness and knowledge about healthy dietary patterns, highlight practices and skills, and present solutions and opportunities to improve food choices.

The development of the messages was guided by best practices related to message length, content, structure, style, tone, framing and wording [19, 21–24]. Message development was also informed by the Theoretical Domains Framework (TDF). The TDF is an integrated theoretical framework that synthesizes 14 domains and 128 theoretical constructs from 33 theories of behavior and behavior change [25]. The domains include: Knowledge, Skills, Social/Professional Role and Identity, Beliefs about Capabilities, Optimism, Beliefs about Consequences, Reinforcement, Intentions Goals, Memory, Attention and Decision Processes, Environmental Context and Resources, Social influences, Emotion, Behavioral Regulation. The TDF encompasses the cognitive, affective, social and environmental influences on behavior and messages were designed to target these domains. Message development went through several iterations that helped refine and simplify the content. In some cases, two or more very similar messages were developed that relayed the same content. This was done intentionally with the aim of identifying the most persuasive message in Stage 5. All messages were developed in Portuguese.

A health communications specialist reviewed all messages to further refine their wording, integrate colloquial terminology without compromising on the precision of the information they communicated, and condensed message length or divided the information communicated into two or more messages to make them simpler to read.

### Step 5: Test of content validity

Messages were tested for their content validity through an online survey created using Qualtrics. This exercise was undertaken to help narrow down the list of messages and to capture feedback on message content and clarity. Messages were presented in the survey in four blocks, organized thematically. A total of 47 experts from across Brazil were invited to give their anonymous feedback on one of four randomly selected blocks. Each block comprised of approximately 10 messages - block one had nine messages, block two comprised of eight messages and blocks three and four had 11 messages each. Experts were chosen from among the contributors listed in the DG, and professors and researchers who had previously published on the DG or were known to use it extensively in their research.

Input was solicited on the importance and clarity of each message [‘In your opinion, how important is the information contained in this message?’; ‘In your opinion how clear is this message?’]. Response options were recorded on a 7-point Likert scale and ranged from very important (or very clear) to not at all important (not at all clear). Suggestions for revisions to the messages were also invited [‘Would you change this message in any way to make it clearer or more convincing? You could add or exclude information or choose to divide the message.’]. Multiple messages on the same theme, with near identical content but different wording were displayed together. Participants were requested to first choose the message (or messages) that they thought were most persuasive and then rate the importance and clarity of each of their choices. Providing feedback on the messages was voluntary and no reimbursement was given to incentivize participation in the 20-min long survey.

Feedback was captured in an Excel spreadsheet and the mean values of message importance and clarity were calculated. Any suggested modifications to message wording were incorporated as appropriate. Since this was a qualitative exercise to help prioritize and shortlist messages, response to questions were compared and messages were selected based on their relative scores and after extensive discussion with members of the study team.

## Results

### Step 1: Content extraction

The content analysis process led to the identification of 63 excerpts from the DG, organized under the following themes: *General recommendations for food choice, Guidelines on how to combine food; The act of eating and commensality;* and *Obstacles in the adherence to recommendations.* The excerpts included content from the NOVA classification, described the advantages of certain NOVA categories over others, and presented information on the frequency, types and combinations of foods to consume. The advantages of regularity of meal times, eating in company and in appropriate environments, the importance of developing culinary skills, strategies for addressing the lack of time and being wary of food marketing, were also captured.

### Step 2: Audience analysis

The results from the audience analysis highlighted barriers to healthy eating that included lack of time (to eat, to cook), difficulty in accessing healthy food, the convenience and the ubiquitous marketing of ultra-processed foods [26]. Irregular meals, multi-tasking while eating, family members eating alone and eating away from home (lunch) were not uncommon [27]. A common belief was that healthy foods were not as flavorful and healthy eating was regarded as a sacrifice. While cooking skills and time needed to cook was generally considered important, buying locally sourced produce, eating together with families and eating less non-vegetarian food was considered less so. Healthy eating was associated with motivated individuals and those with high incomes. Gender-roles were traditionally defined with men contributing financially for food purchases and only cooking during special occasions or when they desired something specific [28–33]. 

### Step 3: Expert review panel

Twenty of the 63 DG excerpts reviewed by the panel were identified as being a priority for message development, after considering the results of the audience analysis. These included introducing the NOVA categorization, focusing on variety in the diet that came from unprocessed food, emphasizing water as the beverage of choice and the importance of creating a healthy eating environment. The panel also suggested creating messages to highlight the role of the home food environment, and alternate forms of meal preparation that did not include frying and to make salient the pleasure of eating. In terms of message organization, the panel recommended presenting messages in the sequence of food-related decision-making to help increase their relevance to daily life and maximize their potential of being relatable and actionable. Besides introducing the NOVA classification and the types of food it contained as an overarching theme, messages were organized around the acts of meal planning, shopping, cooking, and eating. Beyond health gains, developing messages that emphasized environmental sustainability, gender-equality, and cost saving were also suggested based on lessons from the audience analysis.

### Step 4: Message development, theoretical underpinning and message refinement

A total of 111 messages were developed – 20 messages introduced the NOVA categories with examples and suggestions on frequency of consumption, 17 messages targeted planning and organization of meals, 11 focused on shopping and food purchase, 34 messages presented information on cooking and division of labor and 29 conveyed information on the act of eating. In many instances, multiple messages were created, conveying the same information using marginally different wording (see Table 2 for a sample). Messages were short (≤ 3 sentences long), structured to be one-sided, conveyed the most important information at the beginning (anticlimactic), used simple language and were explicit in the information they relayed. They were positive and gain-framed and used an empathetic, solution- or substitution-based tone and were presented in the active voice [24]. Besides raising awareness, the messages focused on goals and skill development, behavior regulation, incentivized positive practices as time and/or cost saving and, in some cases, used social norms to highlight healthy practices as advocated by the TDF [25]. Messages also highlighted gender equity and emphasized pleasure and environmental benefits of adopting certain behaviors.

### Step 5: Test of content validity

A total of 36 experts took the survey to register their feedback on the importance and clarity of the messages. Overall, messages were scored very highly (6.7 for importance and 6.3 for clarity on a scale of 7) and about a fourth of the experts provided detailed feedback on a quarter of the messages. Based on an evaluation of relative scores, 6.5 was decided as the cut-off for shortlisting and messages that scored 6.5 or higher for importance and clarity were selected and further refined based on the detailed suggestions received. This process helped reduce the number of messages from 111 to 40. Table 1 provides an overview of message evolution using two examples.

**Table 1:** The progression of message development from content extraction to message design and refinement using two examples

	Excerpt 1	Excerpt 2
**Content extracted**	Whenever possible, make at least part of your food purchases in markets, fairgrounds, producer fairs, and other places where mostly fresh and minimally processed foods, including organic and agroecological based products, are marketed.	The weakening of the transmission of culinary skills between generations favors the consumption of ultra-processed foods. Whenever possible, cook in company.
**Messages developed**	Are your lists ready? Well, then you are ready to shop! Visit your local markets, fairs, and places where you can buy directly from the producers. You will find fresh and seasonal products at low prices. Shop here to save time and money.	Cook from scratch and prepare your food instead of warming it up from a package. There are many different legumes, whole grains and vegetables to choose from! Try different recipes to add variety and explore all the fresh produce that Brazil has to offer.
	Try shopping at markets, fairgrounds, and producer fairs. Not only would you save money buying foods with less pesticides, you will also be supporting local businesses/family agriculture. Many farmers markets use sustainable farming – your purchases will protect bio-diversity, natural resources. Don’t forget your shopping list!	
		Practice your culinary skills with a friend or family member if you need a small incentive to start. Including your children in the cooking process will stimulate them to acquire important skills.
	More and more Brazilians choose to buy their groceries from local markets and directly from producers, supporting sustainable agricultural practices. Shop at these places to save money and buy food with less pesticides.	Pass on culinary traditions to children! Let them choose the tasks they like best, but make sure they are age-appropriate.
**Messages refined, post content validity**	Are your lists ready? Let’s shop! Visit your local markets, farmers markets or places where you can buy directly from producers. You will find fresh and seasonal foods at lower prices and your purchases will protect bio-diversity, natural resources.	Try making your own food and avoid ready-to-heat meals. There are several varieties of fresh food to choose from. Try different recipes and explore what your region has to offer!
	Brazilians increasingly prefer seasonal and regional foods! In addition to being tastier, they are cheaper and stimulate the local economy.	Cooking can be enjoyable! Practice this skill with friends or family members if you need an incentive to start. Involving your children and making it a fun activity will encourage them to learn how to cook.

The research team extensively discussed suggested revisions and incorporated those that were judged to add more clarity and precision. Some suggestions called for dividing messages further. At the end of the content validation and after incorporating feedback from the experts a total of 40 messages were finalized and tested for face validity with the target population (Step 6 of the study). Nine of these messages had 3–4 variants that communicated nearly identical information. They were retained and tested in Step 6 of the study. Besides incorporating the structural elements described above, the final set of messages presented positive social norms and strategies to economize time, money and effort. The messages went beyond focusing on health benefits and instead highlighted practices that were environmentally sustainable and encouraged gender-equality in tasks. A sample of the messages in English (free translation from Portuguese) is presented in Table 2 and a complete list of the original version in Portuguese is available on request.

**Table 2:** A sample of the behavior-change messages developed based on the Brazilian Dietary Guidelines

Message theme	Message
**Ultra-processed food**	A growing number of Brazilians choose to avoid ‘ultraprocessed foods’. Their means of production, distribution, marketing and consumption damages the environment and interferes with the preservation of food culture.
**Planning and organization of meals**	Plan your meals in advance to help organize food purchases and cooking. Remember: planning saves time, money and effort.
**Shopping and food purchase**	Prioritize fresh, seasonal and local produce. Besides being tastier and cheaper, they help the local economy and are less polluting to transport.
**Cooking and division of labor**	Vegetables are the easiest foods to incorporate into meals. They can be prepared in several ways - eaten raw, or baked, sautéed, or roasted. Try them as salads, soups, or as an accompaniment to rice and beans!
**The act of eating**	Share meal time with family, friends or co-workers, when possible. Eat at the table and try not to be distracted by the TV or your cellphone. Meals times can be very enjoyable!

## Discussion

This study provides the blue-print for the phase-wise development of messages that synthesize the key recommendations of the food-based BDG and communicate the adoption of behaviors and goals that are consistent with it’s message. The creation of messages incorporated evidence-based practices and followed an extensive protocol to ensure appropriateness to the Brazilian context. The multi-step methodology included the use of audience analysis, an expert panel discussion and tested content validity via an online survey. It led to the creation of 111 messages of which 40 were short listed for future testing with the target audience.

The methodology used in this study may easily be adapted to different contexts, while taking into consideration available resources. For instance, due to time and monetary constraints, a review of secondary literature was conducted to get an insight into dietary attitudes, practice and misconceptions among the primary audience of these messages. However, other studies have often used focus group discussions to assess similar constructs or employed a combination of ethnographic observations and in-depth interviews along with focus group discussions [12, 13, 16]. In these studies, the discussions, observations and interviews highlighted the importance of promoting consumer benefits in the messages and providing other reasons for changing dietary patterns besides making the health consequences salient. These methods also provided information on the barriers to achieving successful behaviors that included cost, taste, preference, time, accessibility, lack of motivation, and skills. Planning meals ahead of time, both those eaten at home and outside, and involving the whole family in shopping, and cooking, were some of the strategies suggested [16]. The secondary literature review and the expert panel conducted in this present study, led to the same conclusions. To address similar barriers to healthy practices among Brazilians, the messages made the recommended strategy explicit while highlighting gender equity, and environmental gains and time, effort and cost benefits.

This study incorporated features of the message design to maximize attention and appeal. Messages were therefore concise, direct, simple, gain-framed, positive and, where possible, indicative of a benefit. Besides including recommended elements of messages design, the messages also incorporated important constructs from the TDF – behavior regulation, beliefs about capabilities and consequences, social identity, while presenting skills or goals or sharing knowledge. The messages were also broadly targeted to the free-living, urban, adult Brazilian population and addressed several complex behaviors. Sub-groups of the population that may need special focus may still benefit from these messages but would likely need more customized and tailored communication. Past work has tended to focus on either on one aspect of message design or on a small set of behaviors or a more defined population group [34–39]. These studies differ from the current research in having more focused goals as part of a directed behavior-change intervention. The effectiveness of these messages would therefore be a function of the broader intervention of which they were part.

In the current study, messages were developed to makes the DG more meaningful and easier to put into practice by making explicit the recommended consumer behavior. While no definite platform for dissemination of these messages was finalized, several options were considered that informed their tone, framing and length. These messages would therefore be suitable for presentation via a brochure, or disseminated through a cellphone application, social media or via text messages. They may also be adapted for health campaigns, incorporated into existing health promotion programs or used in conjunction with interventions [40–43]. Future work tests the acceptability of messages among the target population however these messages will have to be implemented as part of a campaign or intervention and evaluated by other studies to assess their effect on actual behavior.

## Conclusions

The present study builds on previous work to develop messages that were not just consistent with the BDG but also appropriate to the context in which they were to be used. It details methods which could be adapted and replicated for message development in other contexts. Integrating methods that allow for a deeper understanding of consumer concerns while providing dietary guidance that motivates them to act, is fundamental to the creation of behavior-change messages.

## Data Availability

The datasets used and/or analysed during the current study are available from the corresponding author on reasonable request.
